# Multiple number-naming associations: How the inversion property affects adults’ two-digit number processing

**DOI:** 10.1177/17470218231181367

**Published:** 2023-07-07

**Authors:** Iro Xenidou-Dervou, Nienke van Atteveldt, Irina M Surducan, Bert Reynvoet, Serena Rossi, Camilla Gilmore

**Affiliations:** 1 Loughborough University, Loughborough, UK; 2 Vrije Universiteit Amsterdam, Amsterdam, The Netherlands; 3 KU Leuven, Leuven, Belgium

**Keywords:** Numerical cognition, number naming, number word transparency, inversion property, audiovisual pairs, bilingualism

## Abstract

Some number-naming systems are less transparent than others. For example, in Dutch, 49 is named “negenenveertig,” which translates to “nine and forty,” i.e., the unit is named first, followed by the decade. This is known as the “inversion property,” where the morpho-syntactic representation of the number name is incongruent with its written Arabic form. Number word inversion can hamper children’s developing mathematical skills. But little is known about its effects on adults’ numeracy, the underlying mechanism, and how a person’s bilingual background influences its effects. In the present study, Dutch-English bilingual adults performed an audiovisual matching task, where they heard a number word and simultaneously saw two-digit Arabic symbols and had to determine whether these matched in quantity. We experimentally manipulated the morpho-syntactic structure of the number words to alter their phonological (dis)similarities and numerical congruency with the target Arabic two-digit number. Results showed that morpho-syntactic (in)congruency differentially influenced quantity match and non-match decisions. Although participants were faster when hearing traditional non-transparent Dutch number names, they made more accurate decisions when hearing artificial, but morpho-syntactically transparent number words. This pattern was partly influenced by the participants’ bilingual background, i.e., their L2 proficiency in English, which involves more transparent number names. Our findings suggest that, within inversion number-naming systems, multiple associations are formed between two-digit Arabic symbols and number names, which can influence adults’ numerical cognition.

## Introduction

Numbers are essential to navigating everyday life. For example, good numeracy skills are linked to positive outcomes in civic engagement and computer literacy (Parsons & Bynner, 2007). However, low numeracy skills are associated with a higher risk of depression and unemployment (Parsons & Bynner, 2005). Consequentially, research on the factors that influence our basic number-processing skills has been flourishing. Cross-cultural research has revealed that one such factor is the linguistic structure of number words (for a review, see Dowker & Nuerk, 2016). For example, in several languages, e.g., Danish, Dutch, German, Malagasy, and Maltese (see Comrie, 2005), the number naming system involves the so-called “inversion property.” That is when the decade-unit order of a number word is the reverse of the written arrangement of its corresponding Arabic symbol (e.g., the Arabic symbol 49 is named “negenenveertig” in Dutch, which translates to “nine and forty”). In essence, with the inversion property, the morpho-syntactic representation of the number name is incongruent with its corresponding written Arabic number representation.

Research has revealed that the inversion property can be problematic for children’s developing numerical skills (e.g., Helmreich et al., 2011; Krinzinger et al., 2011; Miura et al., 1993; Pixner et al., 2011; Steiner, Finke, et al., 2021; Xenidou-Dervou et al., 2015; Zuber et al., 2009). But less is known about its effects on adults’ numeracy, its underlying mechanism, and whether participants’ bilingualism profile moderates its effects. Also, so far, most studies have used between-subject designs (i.e., comparing groups of participants who speak different languages). However, this has the inherent disadvantage of being unable to account for other confounding factors that could explain potential differences between language groups, e.g., cultural, educational, domain-general, or mathematics-specific cognitive differences. The present study aimed to address these gaps by employing a within-subject design where we experimentally manipulated the morpho-syntactic structure of number words to alter their phonological (dis)similarity and numerical congruency with the written Arabic symbol version (see Tables 1 and 2). This allowed us to examine the cognitive mechanism underlying the effects of the inversion property on adults’ basic ability to match the quantity represented by numbers with number names. We also examined whether the participant’s bilingual background influences these effects.

**Table 1.** Design 1: Number words presented for, e.g., the Arabic symbol 49^a^ across the four experimental conditions of the audiovisual matching task.

Arabic symbol: 49		Morpho-syntactic transparency	
		Congruent	Incongruent
Quantity	Match	“*veertig en negen*” (forty and nine)	“negen en veertig” (nine and forty)
	Non-match	“vier en negentig” (four and ninety)	“*negentig en vier*” (ninety and four)

Artificial conditions italicised. Brackets include the English translation of the corresponding Dutch number word.

a See Appendix Table A2 for the complete list of all two-digit numbers used in the audiovisual matching task.

**Table 2.** Design 2: Number words presented for, e.g., the Arabic symbol 49 across the four experimental conditions.

Arabic symbol: 49		Morpho-syntactic transparency	
		Congruent	Incongruent
Quantity	Match	“forty-nine”	“negen en veertig” (nine and forty)
	Non-match	“vier en negentig” (four and ninety)	“acht en twintig” (eight and twenty)

Brackets include the English translation of the corresponding Dutch number word.

### Transparency: linguistic effects on two-digit number processing

Children start learning the verbal counting sequence around the age of 2 (Fuson & Hall, 1983; Gelman & Gallistel, 1978). As development unfolds, children’s number-processing skills evolve and this process is thought to be influenced by cultural innovations including language, writing, and education (Dowker & Nuerk, 2016; Göbel et al., 2011; Ng & Rao, 2010; Xenidou-Dervou et al., 2015). Number-naming conventions across languages differ significantly in terms of transparency. A transparent language is one where two-digit number words reflect the underlying base-10 structure of the numbers they represent. Most Asian languages, for example, have predominantly transparent verbal counting systems, e.g., “ten-three” for 13 (Dowker et al., 2008; Dowker & Nuerk, 2016), whereas the English number-naming system is less transparent (Göbel et al., 2011).

Individuals who speak Asian languages have been found to systematically outperform those who speak less transparent languages in mathematics (e.g., Miura et al., 1993; Ng & Rao, 2010). Although this so-called “Asian advantage” (Mark & Dowker, 2015) may in part be due to educational and cultural differences related to mathematics teaching and learning, this advantage has been observed even before the beginning of formal education (Miller & Stigler, 1987). In 2008, Siegler and Mu showed that Chinese pre-schoolers outperformed their American peers in a number line task, demonstrating performance comparable to that of American children older by one or two years. Similarly, Dowker et al. (2008) showed that compared with English-speaking schoolchildren, speakers of fully transparent Welsh and partially transparent Tamil demonstrated better performance in reading two-digit numbers, comparing two-digit numbers, and conducting arithmetic with two-digit numbers. Therefore, the transparency of number names that we learn may influence the development of our basic numerical skills.

The inversion property is a particular case of non-transparency. Number word inversion appears to influence children’s ability to process two-digit Arabic symbols and perform arithmetic with them. Children who speak languages that incorporate the inversion property exhibit inversion-related difficulties in basic number-processing tasks, such as transcoding (Krinzinger et al., 2011; Pixner et al., 2011; Zuber et al., 2009), symbolic approximate arithmetic (Xenidou-Dervou et al., 2015), as well as exact symbolic arithmetic (Göbel et al., 2014). Inversion seems to also hinder the development of children’s place-value understanding (Moeller et al., 2011), which is an essential building block for the acquisition of more advanced numeracy skills (Okamoto, 2018).

One may expect that the effects of the inversion property would disappear as number names become automatised with age, education, and experience. However, some inversion effects—albeit less pronounced—have also been demonstrated with adult participants. Nuerk et al. (2005) found small inversion-related effects on German-speaking adults’ two-digit symbolic magnitude comparison skills compared with English-speaking adults. Furthermore, Lonnemann and Yan (2015) reported inversion-related effects on adults’ arithmetic performance. They conducted a study in which Chinese- and German-speaking participants heard addition problems with inversed or non-inversed number words in their respective languages (i.e., Chinese-speaking participants heard standard non-inversed Chinese number words and artificially inverted Chinese number words whereas the German-speaking participants heard standard inversed German number words and artificially non-inversed German number words). As would be expected, the Chinese-speaking participants had more difficulties compared with the German-speaking when confronted with inversed number words. Interestingly, however, the German-speaking participants did not encounter more difficulties when hearing the addition problems with non-inversed number words, which essentially were the opposite of the names they had always used. This morpho-syntactic manipulation of the presented number words used by Lonnemann and Yan (2015) provides an excellent method to examine the mechanism through which linguistic characteristics affect mathematical cognition. However, one may argue that in Lonnemann and Yan’s (2015) study perhaps the German-speaking participants’ familiarity with more transparent non-inversed languages, such as English, may have explained the similarity in performance between inversed and non-inversed number words. Surprisingly, to our knowledge, the potential effects that participants’ linguistic background can have on how they process number words have so far not been considered. Crystal (1997; as cited in Bialystok, 2010) estimates that approximately two-thirds of the world’s population is raised in a bilingual environment. Therefore, it is necessary to understand the role of bilingualism in numerical cognition.

To summarise, research so far suggests that the inversion property can influence adults’ arithmetic and potentially even their basic numeracy skills, albeit to a lesser extent than in childhood. But why does this influence persist even in adulthood? To answer this question, we must consider the underlying cognitive mechanisms. It is possible that the morpho-syntactic incongruency inherent in inversed numbers influences the associations made between number names and their corresponding Arabic symbols.

### Associating Arabic symbols to number-names

Associating Arabic symbols to specific number names and vice versa may follow a developmental pathway similar to that of learning the associations between letters and speech sounds (van Atteveldt & Ansari, 2014). There are robust findings in the literature that letters map onto their readily available phonological representations during reading acquisition (e.g., Froyen et al., 2009). Functional magnetic resonance imaging (fMRI) research has shown that speech processing areas in the superior temporal cortex are able to distinguish between letter/speech-sound mappings that are congruent to what we have learned during reading acquisition (e.g., letter symbol “a” presented together with speech sounds /a/) and those with incongruent mappings (letter symbol “o” with speech sound /a/) (van Atteveldt et al., 2004). Noticeably, this ability can predict reading achievement (Blau et al., 2010).

Thus, letter/speech-sound pairs appear to be processed automatically as integrated audiovisual wholes in typically reading adults (Froyen et al., 2009). It is possible that a similar process also unfolds in the case of Arabic symbols and number names, culminating in automatic, integrated audiovisual wholes of Arabic symbol/number-name pairs by adulthood. Holloway et al.’s (2015) fMRI study with English-speaking adults showed that audiovisual single-digit Arabic symbol/number-name pairs elicited a similar congruency effect to that previously found for letter/speech-sound pairs (van Atteveldt et al., 2004). This suggests that with development and education, overlearned associations between symbols and their names can become automatic. But can this assumption be extrapolated to all language groups? Perhaps a lack of number naming transparency, such as in the case of the inversion property described above, alters these associations between symbols and their names.

Furthermore, contrary to letters, numbers carry a crucial extra attribute, the quantities that they represent. According to the Triple Code Model (Dehaene, 1992), numbers are mentally represented in three different codes: the auditory verbal form frame (i.e., the number name), the visual Arabic number form (i.e., the symbol), and the analogue magnitude code (i.e., quantity/amount represented). Alongside notation-specific input–output processes, the Triple Code model proposes internal translation processes between these nodes. These bidirectional translational processes are also often known as transcoding (e.g., Barrouillet et al., 2004; Hayek et al., 2019; Nuerk et al., 2005; Poncin et al., 2020; Salillas & Carreiras, 2013; Steiner, Banfi, et al., 2021).

Several theoretical models of number transcoding have been proposed over the years (e.g., Deloche & Seron, 1987; McCloskey, 1992). However, only one accounts for the development of transcoding, which is the ADAPT model (A Developmental, Asemantic, and Procedural model for Transcoding) put forward by Barrouillet and colleagues (2004). This model starts with the assumption that when an individual hears a verbal string that refers to a number, they store it in their phonological buffer. The level of ease with which we store such a verbal string depends on its length and its phonological dissimilarity from other relevant strings, i.e., how distinguishable this name is from other number names. One of the crucial proposals of the ADAPT model is that when a verbal string that needs to be transcoded corresponds to a representational unit stored in one’s long-term memory (LTM), then its transcription is the result of direct memory retrieval. Retrieval is essentially viewed as a probabilistic process, which depends on the strength of the associations between the verbal string and lexical units stored in LTM; the weaker the strength, the less familiar or rarer the form to transcode. However, if direct retrieval fails, e.g., because the Arabic form is not known (as is the case with young children learning numbers) or the verbal string is not familiar, then, according to ADAPT, a backup strategy is employed known as algorithmic transcoding. When the verbal string cannot be processed as a whole, then the algorithmic strategy initiates a sequential and hierarchical parsing process of the verbal string for transcription by separating the units that can be processed by the production system (Barrouillet et al., 2004). This assumption is especially relevant to multi-digit number processing. To sum up, the ADAPT model would also predict that in adulthood: (1) there would be strong associations between numbers and their names, resulting in the automatic retrieval of number-name pairs; (2) phonological similarities would cause difficulties as they are harder to store in the phonological buffer; and (3) verbal strings that refer to the same representational unit may be co-activated. But what happens in the case where one verbal string is more transparent (dissimilar/easier to distinguish) than the other, as would be the case for Dutch bilinguals who are highly familiar with the Dutch way of naming two-digit numbers that includes inversion as well as the English way which does not and is more transparent?

Furthermore, number names may also automatically activate their respective magnitude representations even if the task does not require one to process the semantic attributes of this number (e.g., Dehaene et al., 1993; Fias et al., 1996). Research on how the magnitude of two-digit numbers is represented suggests three different models: (1) the holistic, which assumes that one maps two-digit numbers onto a single holistic magnitude representation; (2) the decomposition, which assumes that the magnitudes of the two digits in a two-digit number are represented separately; and (3) the hybrid, which assumes that two-digit numbers can be represented both holistically and separately and these two representations may activate or inhibit one another (Nuerk & Willmes, 2005). The nature and generality of magnitude activation during two-digit number processing are controversial and inconclusive (see Nuerk & Willmes, 2005); nevertheless, this literature highlights the fact that the ability to match verbal and Arabic two-digit symbols may interact with quantity representations.

### The current study

The aim of the current study was to examine the effect of the inversion property on adults’ basic numeracy. Specifically, we examined whether Dutch-speaking adults automatically process Arabic symbol/number-name pairs and whether and how the inversion property comes into play in this process. The ability to match symbols with number names is typically assessed with the audiovisual matching task (Sasanguie & Reynvoet, 2014). In this task, participants hear a number word and simultaneously see an Arabic symbol and have to determine whether these are numerically the same. Research has shown that performance on this task predicts arithmetic ability in adult Dutch speakers (Sasanguie & Reynvoet, 2014), suggesting that the ability to fluently translate between digits and number words is important for arithmetic development.

Recently, Poncin et al. (2020) used an audiovisual transcoding design in a study where French and German participants heard a traditional two-digit number word and had to find the matching Arabic two-digit symbol among four presented on the screen with different orders of unit- and 10-digit presentations. This design yielded no negative effect of the inversion on adults’ transcoding speed, which led the authors to conclude that inversion effects disappear by adulthood. Similarly, Steiner, Banfi, et al. (2021) administered to German- and English-speaking adults a number-matching task, where participants had to decide if a spoken number word matched a visual Arabic number and systematically varied the digits in the non-matching distractors. They found that German-speaking adults (inversion language) were slower in rejecting inverted number distractors (e.g., heard “twenty-four,” saw 42) compared with English (non-inversion language). However, both aforementioned studies only manipulated the Arabic digits shown, not the morpho-syntactic structure of the number words that the participants heard and therefore did not examine the phonological processing cost of the morpho-syntactic incongruency of the inversion property. Also, the participants’ linguistic backgrounds were not taken into account, and thus findings may be confounded by the participants’ knowledge of non-inversion languages. To address our research questions, we examined the phonological processing cost imposed by the inversion property by manipulating the morpho-syntactic congruency of number names and the Arabic symbol that they were presented with. Here, morpho-syntactic congruency refers to whether the order of the words in the auditory stimulus matches the decade-unit order of the written Arabic stimulus. To achieve this, we used a modified version of the audiovisual matching task (Sasanguie & Reynvoet, 2014) with two-digit numbers. Participants heard variations of Dutch number words that either matched or did not match the quantity and morpho-syntactic structure of a visually presented Arabic two-digit symbol. This resulted in the 2-by-2 design illustrated in Table 1, which will henceforth be referred to as Design 1.

Design 1 involves four experimental number word conditions (Table 1). The incongruent match cell entails the traditional way of naming “49” in Dutch “negenenveertig” (English translation: “nine and forty”). As described earlier, this number name is morpho-syntactically incongruent with the structure of the Arabic symbol 49. The congruent version of this is: “veertig en negen” (translation: “forty and nine”)—this is still a quantity match, but with an artificial word structure that is congruent with the morpho-syntactic structure of the Arabic symbol (similar to English). In the quantity non-match conditions, the number words now reflect a completely different number. There is the morpho-syntactically congruent non-match condition with the number word “vier en negentig” (translation: “four and ninety”)—that is, the traditional Dutch name for the number 94. Finally, there is the morpho-syntactically incongruent non-match condition with the number word “negentig en vier” (translation: “ninety and four”), which again is an artificial word structure for the number 94 (similar to English). The aim of this systematic morpho-syntactic manipulation was to manipulate the phonological similarity of the number words across match and non-match conditions, e.g., consider the cognitive cost of the phonological similarity between “negen en veertig” (the traditional Dutch name for 49) and “negentig en vier” (the traditional Dutch name for 94”).

As can be seen in Table 1, to achieve this balanced 2-by-2 design we had to use *artificial* number words in the congruent match and incongruent non-match conditions (Italicised in Table 1). Therefore, one could argue that potential morpho-syntactic (in)congruency effects could actually be driven by the artificiality of these conditions. Or perhaps the participants’ knowledge of a non-inversed number naming system—such as English—could influence performance in these conditions. It is commonly known that in the Netherlands people speak English to a high standard and are exposed to the language from a young age in their formal schooling. To address these concerns, we included a second experimental design within this experiment (Design 2—Table 2), which resulted from replacing the two artificial conditions with respective *familiar* number word conditions: the English name of the corresponding Arabic number was used in the quantity match and morpho-syntactically congruent condition and a different Dutch number, without any phonological similarity to the target number, was used in the quantity non-match and morpho-syntactically incongruent condition (see Table 2).

This experimental approach allows us to examine the effect of the inversion property of a number naming system on adults’ numeracy in a within-subject design. This is an important development that overcomes the pitfalls of the cross-cultural designs used in previous research. In within-subject designs, participants serve as their own controls (Greenwald, 1976), thus increasing the likelihood that differences between conditions are due to the experimental manipulation itself rather than individual differences (Keselman & Algina, 1996). This is crucial because cultural and educational differences across cultures are known to play a role in mathematics learning and achievement (e.g., Geary et al., 1996).

Given the findings in the literature on the processing of audiovisual letter/speech-sound pairs (Froyen et al., 2009; Holloway et al., 2015; van Atteveldt et al., 2004), we expected our adult participants to have automatised the Arabic symbol/number-name pairs. Thus, for Design 1, we expected that Dutch-speaking adults would match the Arabic symbols with their corresponding traditional Dutch number name (morpho-syntactically incongruent quantity match) *faster* than the other quantity match condition (*Hypothesis 1*). However, given the literature, which suggests that the inversion property can negatively influence even adults’ basic number-processing accuracy (Nuerk et al., 2005; Lonnemann & Yan, 2015), we expected a morpho-syntactic congruency effect, namely that participants would be more *accurate* in the morpho-syntactically congruent quantity match condition than the other quantity match condition (*Hypothesis 2*) even though this is an artificial number word in Design 1 (Lonnemann & Yan, 2015).

For the quantity non-match conditions in Design 1, predictions were less straightforward because—to our knowledge—such a methodological approach has not been implemented before. The number-names presented in the quantity non-match condition represent different numbers to the target number seen on the screen (e.g., seeing 49 and hearing the name for 94, i.e., four and ninety) but sometimes with phonological similarities (i.e., between “four” and “forty. . .”). Anecdotally, it is well known that people who speak inversion languages, like Dutch, often make inversion mistakes such as saying “94” although they intended to say “49.” If that is the case, then we would expect our participants to find the morpho-syntactically congruent quantity non-match condition (e.g., for the target number 49, they hear the traditional Dutch number-name for the number “94”), to be more challenging to reject (in terms of both RT—*Hypothesis 3a*, and accuracy—*Hypothesis 3b*) compared with the artificial morpho-syntactically incongruent quantity non-match condition, which essentially expresses an addition in Dutch “ninety and four” and is the corresponding artificial way of naming the number “94.” For a summary of all Design 1 hypotheses, see Table 3.

**Table 3.** Summary of hypotheses within Design 1 for the example of the number 49.

Arabic symbol: **49**		**Morpho-syntactic transparency**		**Data**	**Hypothesis**
		**Congruent**	**Incongruent**		
**Quantity**	**Match**	“*veertig en negen*”	**“negen en veertig”**		
		*(forty and nine)*	(nine and forty)		
			X	RT	1
		X		Accuracy	2
	**Non-match**	“vier en negentig”	“*negentig en vier*”		
		(four and ninety)	*(ninety and four)*		
			X	RT	3a
			X	Accuracy	3b

RT: reaction time.

X indicates the condition where we predicted better performance (faster or more accurate).

Design 2 will allow us to determine whether any of the aforementioned effects could be driven simply by the use of artificial number names. As the analyses now included a condition containing English number words, we also had to account for participants’ English-language L2 proficiency. So, for Design 2, overall, we again expected that participants would be faster in the traditional Dutch number word (morpho-syntactically incongruent quantity match) condition than the other quantity match condition, given that Dutch was their first language. But proficient bilingual Dutch-English language speakers may have also automatised the numeral-number-name pairs in English. Given that English number names above 20 have a morpho-syntactically congruent structure, we expected them to be equally fast in the quantity match conditions regardless of whether the number word was morpho-syntactically congruent (English number word) or incongruent (Dutch number word) (*Hypothesis 4*). However, we expected them to be more accurate in the congruent condition (English number word) because of the advantages of morpho-syntactic congruency (*Hypothesis 5*).

For the quantity non-match conditions in Design 2, we expected that participants would be both faster and more accurate in the morpho-syntactically incongruent than the congruent conditions. This is because the morpho-syntactically incongruent condition has absolutely no perceptual and phonological similarity with the name of the target number (e.g., seeing “49” and hearing the name for “28”—“acht en twintig”) and would therefore be easier (*Hypothesis 6a*) and faster (*Hypothesis 6b*) to reject than a number which has some phonological similarity to the target’s number-name. For a summary of all Design 2 hypotheses, see Table 4.

**Table 4.** Summary of hypotheses within Design 2 for the example of the number 49.

Arabic symbol: **49**		**Morpho-syntactic transparency**		**Data**	**Hypothesis**
		**Congruent**	**Incongruent**		
**Quantity**	**Match**	“forty-nine”	**“negen en veertig”**		
			(nine and forty)		
		=		RT High L2 proficiency	4
		X		Accuracy	5
	**Non-match**	“vier en negentig”	“acht en twintig”		
		(four and ninety)	(eight and twenty)		
		X		RT	6a
		X		Accuracy	6b

RT: reaction time.

X indicates the condition where we predicted better performance, L2 = English as Second Language.

To summarise, overall, we expected that phonological similarity (i.e., morpho-syntactic congruency) would be helpful for audiovisual matching when the two-digit Arabic symbols and number word match quantity-wise, but unhelpful when they do not. However, experience with another more transparent language may also play a role.

## Method

### Participants

Sixty adult participants aged 19.41 to 65.33 years (*M* = 30.75; *SD* = 14.72) took part in the experiment. Participants were recruited through social media and word-of-mouth. More than half of the sample consisted of university students (*N* = 33; 55%) attending a full-time or part-time course. Among the total sample, 45 (75%) had a paid job or volunteered at the time of the participation, while the remaining sample (*N* = 15; 25%) were unemployed, retired, or did not specify their employment status. None of the participants had a learning disability or visual or auditory impairment. After reviewing participants’ responses to the Language Experience and Proficiency Questionnaire (LEAP-Q; Marian et al., 2007), one participant was excluded from the analysis, as Dutch was not their first language, and another because English was not their second language. We also checked for outliers in the accuracy of the Audiovisual matching task using the ±3 standard deviations from the mean criterion and found no extreme outliers. In the case of the RT data, we used the median scores, which are not affected by outliers. The final sample consisted of 58 (35 females) Dutch-English bilinguals. Based on self-reported measures in the LEAP-Q questionnaire, the average age of acquisition of English across participants was 11 years (*SD* = 2.3) and the average age they became fluent in English 14 years (*SD* = 2.6). All participants received 10€ compensation for their time. Written informed consent was obtained from all participants. The study was conducted in accordance with the ethical guidelines of the Vrije Universiteit Amsterdam.

### Procedure

Trained experimenters tested each participant individually in a quiet setting on all tasks besides the LEAP questionnaire. This was sent to the participants via email prior to the testing day; they were asked to fill it in and send or bring it with them on the day of testing. The data presented in this article comprise participants’ performance on a subset of tasks administered by the experimenters as part of a larger unpublished project. The entire testing session with each participant lasted approximately 1 hr.

### Materials

#### The audiovisual matching task

The audiovisual matching task was presented in E-prime (Psychology Software Tools Inc., Pittsburgh, PA) on an HP ProBook 6650b laptop. This was an adapted version of the audiovisual task used by Sasanguie and Reynvoet (2014). The participants saw an Arabic target number on the screen and simultaneously heard a number word. They were instructed to decide whether the number-name that they heard matched the quantity of the Arabic number that they saw or not.

First, the participants were given six practice trials with two-digit numbers, which were not used in the testing trials, to become familiar with the requirements of the task. Each trial started with a fixation cross displayed for 500 ms in the centre of the screen, followed by the simultaneous presentation of a visual Arabic symbol and a number word. The Arabic symbol remained on the screen until a response was made via key press, indicating whether the Arabic symbol and the number word matched or not (keys q or p, respectively). Participants only received feedback on the practice trials.

The visual and auditory stimuli consisted of two-digit Arabic symbols ranging from 24 to 98 in six experimental conditions (Design 1/Table 1 and Design 2/Table 2). Table A2 in the online Supplementary Material includes all the testing trials presented within the audiovisual matching task. The task included six blocks of trials. Each block included 24 trials pooled from all 6 experimental conditions—one trial from each Arabic number target, e.g., Block 1 included the 24 trials highlighted in grey in Table A2. In 50% of the trials, the number word matched the quantity of the Arabic two-digit symbol, and in the remaining 50%, it did not. All blocks were presented twice, and trial order was randomised within each block.

Accuracy was computed by summing up all correct responses in each experimental condition. RT measurement of each trial started with the onset of the presentation of the visual and auditory stimuli until a response was made via keypress by the participant. We deliberately opted for our participants to be able to respond from the moment they heard and saw the stimuli as this is more ecologically valid and it increases the sensitivity of our experimental design to detect effects on both RT and accuracy.

The auditory stimuli were recorded by a native female Dutch speaker in Audacity® (www.audacityteam.org), at a frequency of 22.05 kHz and had a duration of 800–1,400 ms. The participants heard the auditory stimuli through Sennheiser HD201 headphones and responded using the laptop’s integrated keyboard. All audio recordings as well as a video recording illustrating how the audiovisual task worked are available in an Open Science Framework (OSF) directory: https://osf.io/f2xwd.

#### The LEAP-Q

Participants’ language profiles were assessed with the Dutch translation of the LEAP-Q (Marian et al., 2007). This questionnaire has been constructed based on bilingualism theories that view L2 (second language) acquisition as an interaction between language proficiency and experience variables, and it is known to be a reliable and valid questionnaire for assessing adults’ bilingual status (Marian et al., 2007). In the present study, this questionnaire was used to: (1) screen for people who had Dutch as their first language and English as their second (see section “Participants”) and (2) to control for participants’ English proficiency when analysing the conditions that included English number words.

A covariate “English proficiency” was computed for each participant, based on the English competence factor identified by Marian et al. (2007; Study 1). Specifically, the items that loaded onto the English competence factor [Exposure to English (% time), exposure to TV in English (scale 0–10), exposure to radio in English (scale 0–10), exposure to family in English (scale 0–10), preference to speak in English (%), exposure to reading in English (scale 0–10), exposure to English classes/self-instruction (scale 0–10), and learning English from reading (scale 0–10)] were standardised and added together in a composite score.

## Results

This section is divided into two parts. First, we report the results of RT and accuracy analyses for Design 1 (see Table 1) to address Hypotheses 1–3 (Table 3). We then report the results of RT and accuracy analyses for the Design 2 conditions (Table 2) to address Hypotheses 4–6 (Table 4). Anonymised Datasets, figures, and analyses for R scripts are available in the OSF (https://osf.io/f2xwd).

### Effects of number word morpho-syntactic congruency (Design 1)

**Figure 1.**
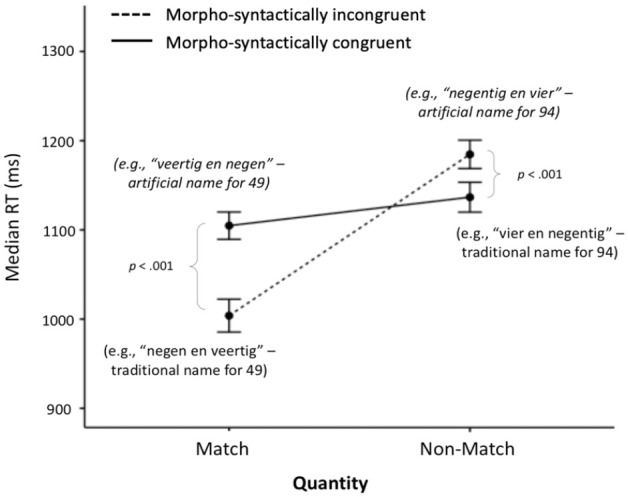
Median RT ± 1 SEM in the Design 1 audiovisual conditions. For ease of understanding, in brackets, we give the meaning of the corresponding number word that participants heard in each condition for the Arabic target number “49” (Figure available at https://osf.io/f2xwd, DOI: 10.17605/OSF.IO/F2XWD, under a CC-BY4.0 licence).

To test Hypotheses 1 and 3a, we ran a quantity match (2 levels: quantity match, non-match) × morpho-syntactic congruency (2 levels: congruent, incongruent) analysis of variance (ANOVA) on median RT of the correct trials for the four conditions in Design 1 (see Table 1). Results demonstrated main effects of quantity matching, *F*(1, 57) = 109.51, *p* < .001, $\eta_{p}^{2}=.66$ , morpho-syntactic congruency, *F*(1, 57) = 16.4, *p* < .001, $\eta_{p}^{2}=.22$ , and the predicted interaction, *F*(1, 57) = 65.93, *p* < .001, $\eta_{p}^{2}=.54$ (see Figure 1), which revealed that the effects of the morpho-syntactic congruency were different between the match and non-match conditions.

This interaction was further examined by running an ANOVA separately for each level of quantity matching. In the quantity match condition, there was a significant difference between the morpho-syntactically congruent and incongruent trials, *F*(1, 57) = 102.24, *p* < .001, $\eta_{p}^{2}=.64$ . As predicted, participants were significantly faster in the condition where they heard the traditional Dutch number name compared with the artificial one (Figure 1), even though the phonology of the artificial name matched the structure of the Arabic symbols. This finding supports *Hypothesis 1*. We also found an effect of transparency in the quantity non-match trials, *F*(1, 57) = 15.01, *p* < .001, $\eta_{p}^{2}=.21$ (Figure 1), albeit the opposite of what we expected with *Hypothesis 3a*. Namely, we found that participants were slower in the quantity non-match trials when the number name was morpho-syntactically incongruent rather than congruent with the target number (e.g., 49). In other words, contrary to our prediction, they were faster to recognise that the traditional Dutch name for “94” (vier en negentig, which in English translates to “four and ninety”) does not match the Arabic symbol “49” that they saw on the screen, compared to hearing the artificially non-inverted number name for “94” (i.e., “negentig en vier,” which in English translates to “ninety and four”).

**Figure 2.**
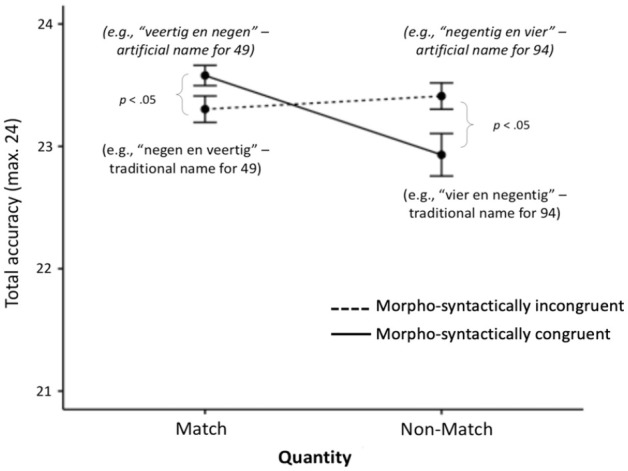
Accuracy ± 1 SEM in the Design 1 audiovisual conditions. For ease of understanding, in brackets, we give the meaning of the corresponding number word that participants heard in each condition for the Arabic target number “49” (Figure available at https://osf.io/f2xwd, DOI: 10.17605/OSF.IO/F2XWD, under a CC-BY4.0 licence).

To address *Hypothesis 2 and 3b*, we ran an ANOVA for Design 1’s accuracy data this time. There were no main effects for quantity matching or morpho-syntactic congruency. However, as expected, there was a significant quantity matching × morpho-syntactic congruency interaction, *F*(1, 53) = 8.44, *p* = .005, $\eta_{p}^{2}=.14$ (see Figure 2). This interaction was further examined by running an ANOVA separately for each level of quantity matching. For match trials, there was a significant effect of morpho-syntactic congruency, *F*(1, 54) = 4.71, *p* = .034, $\eta_{p}^{2}=.08$ , which showed that participants were more accurate in the morpho-syntactically congruent condition (e.g., Artificial name for 49) than in the incongruent condition (e.g., Traditional name for 49). This supports *Hypothesis 2*, namely, our Dutch-speaking participants benefitted from a morpho-syntactically congruent number word structure, even though it was artificial, and they have been naming the numbers in the reversed manner their entire lives.

There was also a significant effect of morpho-syntactic congruency for non-match trials, but as expected in the opposite direction, *F*(1, 55) = 6.28, *p* = .015, $\eta_{p}^{2}=.10$ (Figure 2). Specifically, participants performed better when the number name was morpho-syntactically incongruent with the target number, e.g., when they saw 49 and heard an artificial name for 94. In other words, they made more mistakes in the condition where they heard the traditional name for 94 in line with *Hypothesis 3b*.

### The role of word familiarity and English language proficiency as an L2 (Design 2)

To test Hypotheses 4–6, we now used the conditions in Design 2 (see Tables 2 and 4), first with RT and then accuracy data.

**Figure 3.**
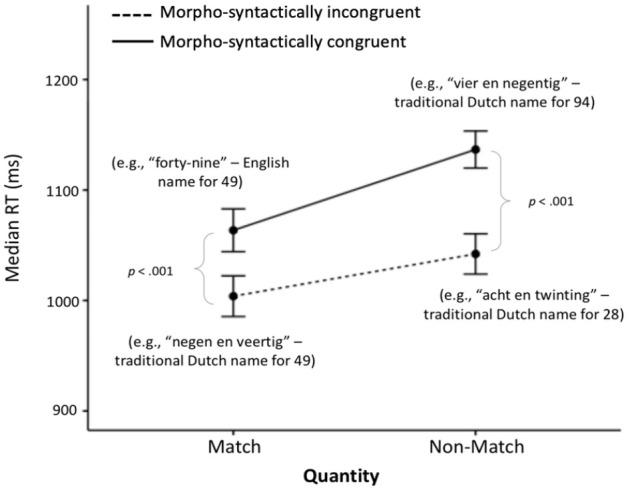
Median RT ± 1 SEM on the Design 2 conditions. For ease of understanding, in brackets, we give the meaning of the number word that participants heard in the corresponding condition (Figure available at https://osf.io/f2xwd, DOI: 10.17605/OSF.IO/F2XWD, under a CC-BY4.0 licence).

For Hypotheses 4 and 6a, a quantity matching (2 levels: matching, non-matching) × morpho-syntactic congruency (2 levels: congruent, incongruent) analysis of covariance (ANCOVA) was run on median RT for the Design 2 conditions, with English proficiency as a covariate. Once again, the ANCOVA showed a significant main effect of quantity matching, *F*(1, 56) = 42.84, *p* < .001, $\eta_{p}^{2}=.43$ , morpho-syntactic congruency, *F*(1, 56) = 105.01, *p* < .001, $\eta_{p}^{2}=.65$ , and their interaction, *F*(1, 56) = 4.88, *p* = .031, $\eta_{p}^{2}=.08$ (see Figure 3), but there was no significant interaction with the covariate on English proficiency.

The observed quantity matching × morpho-syntactic congruency interaction was examined by conducting an ANOVA separately for each level of quantity matching. For the match trials, contrary to *Hypothesis 4*, there was a significant effect of morpho-syntactic congruency, *F*(1,57) = 31.44, *p* < .001, $\eta_{p}^{2}=.36$ , with participants being significantly faster in the morpho-syntactically incongruent condition (i.e., the traditional Dutch way of naming the given number) than in the congruent condition (i.e., the corresponding English number name). There was also a significant effect of transparency for the non-match trials, *F*(1, 57) = 74.28, *p* < .001, $\eta_{p}^{2}=.57$ , which showed that, as per *Hypothesis 6a*, participants were significantly faster in the morpho-syntactically incongruent condition (i.e., the traditional Dutch name of a phonologically completely different two-digit number) than in the morpho-syntactically congruent condition (i.e., the inverted number compared to the target, i.e., they saw 49 and heard the Dutch number word for ninety-four).

To test Hypotheses 5 and 6b, we now ran a quantity matching (2 levels: matching, non-matching) × morpho-syntactic congruency (2 levels: congruent, incongruent) ANCOVA on accuracy data for the Design 2 conditions, and English proficiency as a covariate. Results demonstrated a main effect of morpho-syntactic congruency, *F*(1, 52) = 12.32, *p* = .001, $\eta_{p}^{2}=.19$ , the expected quantity matching × morpho-syntactic congruency interaction, *F*(1, 52) = 12.02, *p* = .001, $\eta_{p}^{2}=.18$ , and this time also a significant quantity matching × morpho-syntactic congruency × English proficiency interaction, *F*(1, 52) = 7.88, *p* = .007, $\eta_{p}^{2}=.13$ . To elucidate this three-way interaction, a median split was performed on the L2 English proficiency scores (LEAP questionnaire) to obtain a variable with two levels—low and high English proficiency. Descriptive statistics of each item included in the LEAP questionnaire and the composite (*z*-score) in the High and the Low English proficiency group are reported in Table 5. It is worth noting that the comparison between the High proficiency group and the Low proficiency group in the composite score of the LEAP questionnaire showed a significant difference, *t*(56) = 7.99, *p* < .001, *d* = 2.10.

**Table 5.** Descriptive statistics of the items included in the LEAP questionnaire and the composite score (*z*-score) in the High and Low proficiency groups separately.

LEAP item	Low proficiency group	High proficiency group
Exposure to English (% time)	9.69 (7.56)	22.28 (12.10)
Exposure to TV in English (0–10)	5.79 (2.47)	7.45 (1.64)
Exposure to radio in English (0–10)	5.72 (2.79)	6.59 (2.38)
Exposure to family in English (0–10)	0.55 (1.70)	2.28 (3.13)
Preference to speak English (%)	3.10 (6.47)	11.62 (17.74)
Exposure to reading in English (0–10)	4.55 (3.00)	7.69 (1.44)
Exposure to self-instruction (0–10)	2.41 (3.25)	6.76 (2.57)
Learning English from reading (0–10)	5.59 (2.37)	7.93 (1.33)
Composite score (*z*-scores)	−0.42 (0.42)	0.42 (0.38)

LEAP: Language Experience and Proficiency.

Means (standard deviations) of the raw scores of the items used for creating the composite score (L2 proficiency).

**Figure 4.**
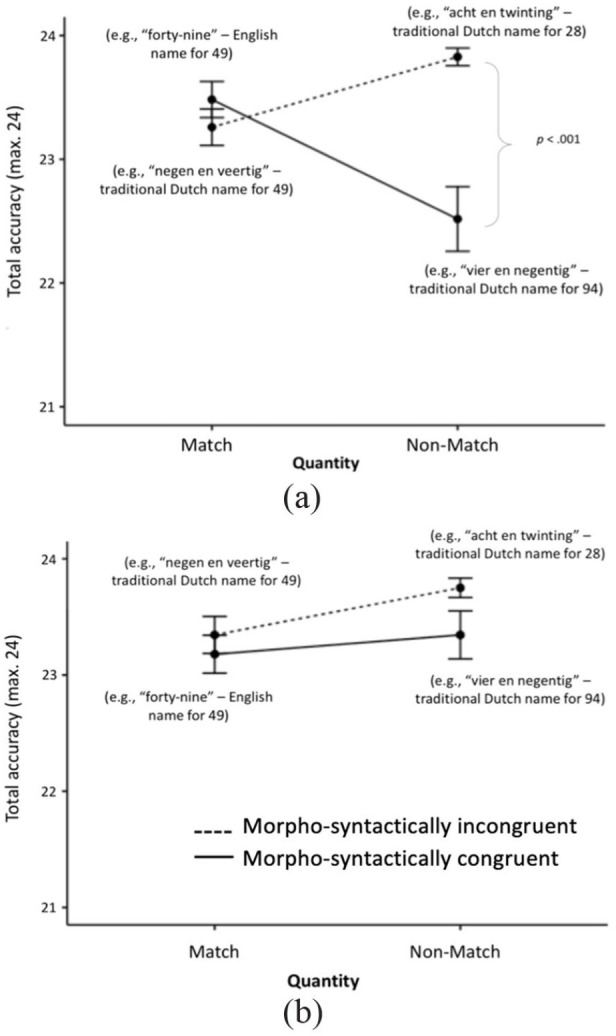
High (a) and Low (b) English proficiency participants’ accuracy in the Design 2 conditions ± 1 SEM. For ease of understanding, in brackets, we indicate the meaning of the number word that participants heard in the corresponding condition (Figure available at https://osf.io/f2xwd, DOI: 10.17605/OSF.IO/F2XWD, under a CC-BY4.0 licence).

A quantity matching × morpho-syntactic congruency ANOVA was then run separately for each level of English proficiency. For low English proficiency, the ANOVA revealed no effect of quantity matching, morpho-syntactic congruency, or their interaction. Thus, contrary to *Hypothesis 5*, for Dutch speakers with low English proficiency, morpho-syntactic transparency did not appear to affect their audiovisual matching accuracy. For high English proficiency, the ANOVA revealed a main effect of morpho-syntactic congruency, *F*(1, 26) = 8.92, *p* = .006, $\eta_{p}^{2}=.26$ , and a significant quantity matching × morpho-syntactic congruency interaction, *F*(1, 26) = 19.16, *p* < .001, $\eta_{p}^{2}=.42$ (see Figure 4). The interaction was further examined by conducting a separate ANOVA at each level of quantity matching.

For the match trials, contrary to *Hypothesis 5*, there was no effect of morpho-syntactic congruency. Participants performed just as accurately in the morpho-syntactically congruent English condition as they did in the incongruent Dutch condition. However, as expected with *Hypothesis 6b*, we found a morpho-syntactic congruency effect for the non-match trials, *F*(1, 28) = 22.41, *p* < .001, $\eta_{p}^{2}=.45$ . Participants highly proficient in English were significantly more accurate in the morpho-syntactically incongruent Dutch condition (i.e., when they heard the name of a completely different number) than in the morpho-syntactically congruent Dutch condition (Figure 4a). Table 6 shows a summary of descriptive statistics across all experimental conditions, and Table 7 summarises all results on RT and accuracy data across both Designs.

**Table 6.** Summary of descriptive statistics across all conditions of the audiovisual matching paradigm.

E.g. Arabic symbol: **49**		**Design 1**					**Design 2**				
		**Morpho-syntactic transparency**				**Data**	**Morpho-syntactic transparency**				**Data**
		**Congruent**		**Incongruent**			**Congruent**		**Incongruent**		
**Quantity**	**Match**	“*veertig en negen*” (forty and nine)		“negen en veertig” (nine and forty)			“forty-nine”		“negen en veertig” (nine and forty)		
		*M* 1,104.73	*SD* 116.36	*M* 1,003.88	*SD* 140.34	RT	*M* 1,063.55	*SD* 147.41	*M* 1,003.88	*SD* 140.34	RT
		23.58	0.62	23.30	0.81	ACC	23.33	0.83	23.30	0.81	ACC
	**Non-match**	“vier en negentig” (four and ninety)		“*negentig en vier*” (ninety and four)			“vier en negentig” (four and ninety)		“acht en twintig” (eight and twenty)		
		*M* 1,136.65	*SD* 127.59	*M* 1,184.69	*SD* 121.15	RT	*M* 1,136.65	*SD* 127.59	*M* 1,042.13	*SD* 138.98	RT
		22.93	1.32	23.41	0.8	ACC	22.93	1.32	23.79	0.41	ACC

RT: reaction time; ACC: total accuracy.

Brackets include the English translation of the corresponding Dutch number word. Artificial conditions italicised.

**Table 7.** Summarised representation of results across Designs 1 and 2 of the audiovisual matching paradigm.

E.g. Arabic symbol: **49**		**Design 1**			**Design 2**		
		**Morpho-syntactic transparency**		**Data**	**Morpho-syntactic transparency**		**Data**
		**Congruent**	**Incongruent**		**Congruent**	**Incongruent**	
**Quantity**	**Match**	*“veertig en negen”* (forty and nine)	“negen en veertig” (nine and forty)		“forty-nine”	“negen en veertig” (nine and forty)	
			X	RT		X	RT
		X		ACC	=		ACC
	**Non-match**	“vier en negentig” (four and ninety)	*“negentig en vier”* (ninety and four)		“vier en negentig” (four and ninety)	“acht en twintig” (eight and twenty)	
		X		RT		X	RT
			X	ACC		X	ACC High L2
					=		ACC Low L2

RT: reaction time; ACC: total accuracy.

Brackets include the English translation of the corresponding Dutch number word. Artificial conditions italicised. X indicates the match or non-match condition where participants performed better.

We also checked whether a speed–accuracy trade-off explained our results by running a series of correlations between each experimental condition’s accuracy and RT data in both Designs (1 and 2). Specifically, the correlations between accuracy and RT across conditions ranged from −0.06 to 0.21, and none of them were significant. These results suggest that there was no speed–accuracy trade-off in any of our experimental conditions.

## Discussion

The aim of the current study was to examine whether and how the inversion property of a number naming system, i.e., where the unit is named first, followed by the decade (e.g., in Dutch, 49 is named “negenenveertig,” which translates to “nine and forty”) affects adults’ two-digit number processing. Dutch-English bilingual adults performed an audiovisual matching task where they saw a two-digit Arabic symbol, simultaneously heard a number word, and simply had to decide if these matched in quantity. In Design 1 of the experiment, we experimentally manipulated the phonological similarity of the number words and Arabic symbols by manipulating the morpho-syntactic structure of the auditory words so that they were either congruent or incongruent with the place-value structure of their Arabic symbol counterparts (see Table 1 for design and Table 7 for a summary of the findings). Although participants were faster in making decisions when they heard the traditional Dutch number names, which are non-transparent (with inversion), they were more accurate when they heard transparent artificial number words (without inversion).

**Figure 5.**
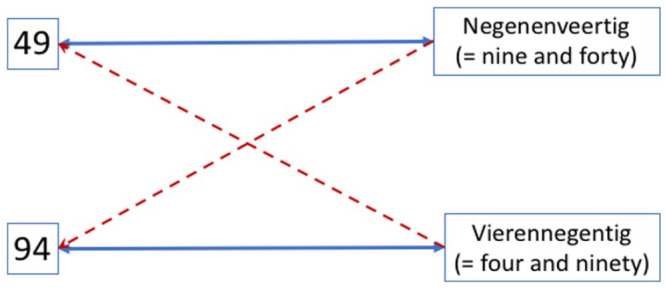
Multiple number-naming associations. Hypothesised theoretical model on how the inversion property affects numeracy. Our findings suggest that the “inverted associations” (depicted with dashed lines) are stronger for bilinguals highly proficient in a second language that involves a more transparent number-naming system (Figure available at https://osf.io/f2xwd, DOI: 10.17605/OSF.IO/F2XWD, under a CC-BY4.0 licence).

With Design 2, we examined whether the previous results could be attributed to the artificiality of the number words and whether participants’ proficiency in English as a second language, which is more transparent compared with Dutch (no inversion for two-digit numbers above 20), played a role (see Table 2 for design and Table 7 for a summary of the findings). Once again, participants were faster in the conditions that involved traditional Dutch number names despite these being non-transparent, further strengthening the assumption that adults have automatised this way of naming two-digit numbers. But when it comes to accuracy, results showed that Design 1 findings were partly influenced by the participants’ existing English language knowledge. For quantity match trials, they were equally as accurate in the condition where they heard the English number-names (without inversion) as when they heard the Dutch number-names (with inversion)—despite English only being their second language. For quantity non-match trials though, results differed on the basis of the participants’ proficiency. For those with low English proficiency, morpho-syntactic transparency did not play a role, when they saw, e.g., 49, they were as accurate in deciding a non-match when hearing the number names 94 as hearing the name for 28. However, those with high L2 English proficiency were more influenced by the morpho-syntactic non-transparency due to the inversion. These participants were thus more accurate in the condition where they heard a number name that had no phonological similarity whatsoever with the name of the target number. Thus, it appears that in non-transparent number-naming systems that include the inversion property, multiple associations are formed between Arabic numbers and number names (Figure 5). Incorrect associations between Arabic symbols and number names may be stronger for those who are highly proficient in a second language, especially a more transparent one than one’s native language. Below we discuss our findings and respective theoretical implications in more detail.

Number-naming transparency is mostly considered an issue in the early stages of number development. As described earlier, there is robust evidence that the inversion property can create difficulties for children (e.g., Helmreich et al., 2011; Krinzinger et al., 2011; Miura et al., 1993; Pixner et al., 2011; Xenidou-Dervou et al., 2015; Zuber et al., 2009). Adults, however, are capable of advanced mathematics and have used numbers and their names so much throughout the years that one may not intuitively consider number names as a potentially influential factor. Especially when it regards the simple task of matching Arabic numbers to their names, it should be as easy as matching a letter to its sound. Our study, however, suggests that it is not as simple as that.

Numbers and letters are similar in a way—they are both culturally invented symbols (van Atteveldt & Ansari, 2014). Research on learning letters and speech sounds suggests that, in adulthood, letter/speech-sound pairs are processed automatically as integrated audiovisual wholes (Froyen et al., 2009). Thus, we hypothesised that similar associations may be formed between numbers and their names during mathematics learning and that by adulthood Arabic symbol/number-name pairs will also be processed automatically as integrated audiovisual wholes (Holloway et al., 2015). Indeed, in both designs of our audiovisual paradigm, participants were faster when hearing the traditional Dutch number names, despite the inherent inversion property (Table 7). This suggests that strong associations have been drawn between numbers and their names throughout the years (e.g., in Figure 5, between 49 and its name “negenenveertig,” and between 94 and its name “vierennegentig”).

However, numbers and letters also have a fundamental difference—the first also reflect quantity (Dehaene, 1992). In both designs, both with the RT and accuracy data, we found an interaction of morpho-syntactic transparency (congruent or incongruent) with quantity (match or non-match). Namely, morpho-syntactic transparency affected quantity match and quantity non-match decisions differently. This was particularly clear in Design 1 (see Table 7). With the RT data, where we saw the automatised—albeit morpho-syntactically incongruent—number names taking the lead in making quick quantity-match decisions, we found the direct opposite facilitating quick quantity non-match decisions, i.e., morpho-syntactic congruency. So, when our Dutch-speaking participants had to match a two-digit Arabic symbol and a number word that represented the same quantity, they were faster when hearing the traditional Dutch number word, e.g., seeing 49 and hearing “negen en veertig” (nine and forty) which includes the inversion property. However, when the two-digit Arabic symbol and the number word did not represent the same quantity, participants were faster at rejecting the match when they heard the familiar Dutch number word for the inverted number, e.g., “vier en negentig” (four and ninety) rather than the unfamiliar “negentig en vier” (ninety and four). In this case, besides “vier en negentig” being a familiar number word and thus the automatised Dutch name of the number 94, it also has the most phonological or perceptual dissimilarity with the traditional name for 49, “negen en veertig”—their onset is different and there is no “vier” in the latter. This means that it can be quickly rejected without even needing to hear the entire number word (although there is the symbol 4 (“vier) in the visual Arabic symbol). Similarly, Steiner et al.’s (2021) findings have previously highlighted the importance of perceptual (dis)similarities in audiovisual matching. In our case here, familiarity and phonological dissimilarity overlap, making it difficult to distinguish between them. However, accumulating our findings across the match and non-match conditions across both Designs (see Table 7), we see that when it comes to RT, familiarity plays a prominent role circumventing the obstacle of in-transparency. In line with the predictions derived from the ADAPT model (Barrouillet et al., 2004), we find that stronger associations appear to be formed with familiar number words and in this case, the confidence for rejecting the audiovisual match is further amplified by the lack of phonological similarity with the automatised correct Dutch name of the target number.

The reversed pattern occurred with the accuracy data; the morpho-syntactically congruent artificial number words facilitated more accurate quantity-match decisions, whereas morpho-syntactic incongruency facilitated more accurate non-match decisions. The latter is a clear illustration of the way in which number-naming transparency causally influences numerical cognition even after years of practice and experience; the more congruent the morpho-syntactic structure of a number word is with the structure of its Arabic symbol, the better one’s ability to match a number with its name (see also Hayek et al., 2019; Lonnemann & Yan, 2015; Nuerk et al., 2005). Design 2 results further clarified the interactive relationship between morpho-syntactic (in)transparency and quantity matching. In Design 2, we replaced the unfamiliar artificial number words from Design 1 with familiar versions, which are more ecologically valid. We had initially hypothesised that proficient bilinguals would have also automatised the corresponding English number words and would, therefore, be equally as fast when hearing them in Dutch or English, and more accurate in the English condition because it does not involve inversion. Interestingly, results demonstrated a different pattern of results. Participants were shown again to be faster in matching quantities when hearing the Dutch number words, no matter their bilingual proficiency level (Figure 3). Thus, two-digit number words in one’s first language appear to be more overlearned and automatised compared with those of an L2, overshadowing the lack of transparency (Holloway et al., 2015). Alternatively, this result may also be influenced by the fact that only one condition contained English stimuli and were therefore relatively infrequent compared with Dutch stimuli. However, this is unlikely because participants were slowest in a Dutch-word condition, namely when they heard the traditional Dutch name for the inverted number word (Figure 3). Nevertheless, future research should consider balancing the number of stimuli across the two languages. When it came to accuracy, participants were equally accurate in the Dutch and the English condition. This is interesting because although they did not perform better in the English condition, as per our inversion hypothesis, they also did not perform worse, despite Dutch being the automatised way of naming two-digit numbers. It would be interesting to replicate this study in different samples and with different distractors and bilingual backgrounds, e.g., with another non-transparent language that includes the inversion as L1 and an even more transparent language as L2 (Göbel et al., 2011) to test how far familiarity interacts with transparency.

In Design 2, the effects of morpho-syntactic non-transparency, the consequent phonological (dis)similarity, and the role of bilingualism herein were further clarified with results in the quantity non-match conditions. As expected, participants were faster in deciding that an auditory number word does not match an Arabic symbol when this word was not only morpho-syntactically incongruent but also had no phonological and perceptual similarity whatsoever. Within our task’s design, we deliberately opted for our participants to be able to respond from the moment that the auditory and visual stimuli were presented for the sake of ecological validity. This is what happens in real life. Consider, e.g., when you are at the counter and you hear the amount from the cashier and see it on the checkout counter, or when you watch the news or attend a conference and you see numbers on graphs and hear the numbers being described from the presenters (sometimes mistakenly incorrectly or inverted). In such real-life circumstances, no one forces one to hear the entirety of a number word and thus errors are likely to occur. When the number word one hears is completely dissimilar from the target visual Arabic symbol from the get-go, i.e., from the first morphemes of a word, then a faster—with almost no cognitive cost—non-match decision can be made. This is in line with the ADAPT model’s assumption that the level of ease with which a verbal string is stored in one’s phonological buffer depends on its phonological dissimilarity from other relevant verbal strings, in our case the level of dissimilarity of one two-digit number name from another. It would be interesting for future studies to examine if and how results change when participants must fully process the auditory stimuli within this audiovisual paradigm.

We observed similar results for the high L2 proficiency participants’ accuracy but, surprisingly, not for those who were not so proficient in English. In other words, our results showed that the Dutch bilinguals who were highly proficient in English made more mistakes in the condition where they saw the number 49 and heard “vier en negentig” (see Figure 4). To our knowledge, this is the first time where we see the differential effect of the inversion property based on one’s level of bilingualism with a more transparent number naming L2. With both experimental designs, hearing the inverted number name to the target Arabic symbol, e.g., seeing 49 and hearing the name for 94, had an effect on participants’ accuracy. These are completely different numbers with a large magnitude difference, which should not be confused. Yet their phonological similarity seems to be an influential factor.

Cumulatively, these findings suggest that for inversion languages, besides the primary strong association between a two-digit Arabic symbol and its name, an additional association can be formed. This extra association is between the inverted version of the target number name, which is not a quantity match but is phonologically similar due to the morpho-syntactic congruency aspect to it that complicates things (see Figure 5). This “inverted association” appears to be stronger for those highly proficient in a more transparent language that does not involve inversion, like English (at least for numbers above 20). Thus, as our results suggest, inversion errors can mistakenly occur when you are very fluent in a non-inverting L2 and thus have formed stronger associations between the Arabic symbol and the morpho-syntactically congruent (more transparent) way of naming the number.

Current theoretical models on bilingualism suggest that bilinguals activate both languages when processing words and that they can share representations across languages, such as syntax, orthography, and phonology (for reviews, see Brysbaert & Duyck, 2010; Li & Xu, 2022). Recent computational models have also been incorporating the dynamic interaction between an individual’s L1 and L2 and thus accounting for L2 proficiency and viewing bilingual language learning as a dynamic, interactive, and evolving process (Li & Xu, 2022). The question of how bilinguals process numbers is another ongoing debate (e.g., Macizo et al., 2011a, 2011b; Spelke & Tsivkin, 2001a, 2011b). Macizo et al., (2011a, 2011b) gave German/English bilinguals with high and low L2 English proficiency an Arabic two-digit number comparison task and found L2 proficiency to have no effect. However, in their second experiment, high and low L2 German/English bilinguals were given verbal comparison tasks in German and English. This time L2 proficiency influenced results; the less proficient bilinguals demonstrated the typical compatibility and incompatibility effects in German and English, respectively (as one would expect from monolinguals), whereas the high L2 bilinguals did not show any such effect. The authors concluded that this finding suggests that bilinguals with lower L2 proficiency are more vulnerable to influences of the language in which numbers are presented and that overall L2 proficiency influences two-digit number processing. Our results extend this field of research by confirming that L2 proficiency influences two-digit number processing also in audiovisual tasks and that high L2 bilinguals are more vulnerable to making accuracy errors when their L2 has a more transparent morpho-syntactic number word system to their L1. To our knowledge, our study is the first to demonstrate the effects of the dynamic interaction of bilinguals’ L1 and L2 in two-digit verbal number processing.

The evident bidirectional internal translation process between visual (Arabic symbol) and auditory input (number word) is in line with the Triple Code Model for numerical cognition (Dehaene, 1992). Our findings extend this model by suggesting that in non-transparent number-naming languages which involve the inversion property, the visual Arabic and the auditory verbal nodes of a given two-digit number may also be associated with the nodes of the inverted number as depicted in Figure 5, which may lead to the inversion errors that we observe even in adulthood (Nuerk et al., 2005; Lonnemann & Yan, 2015).

Steiner, Banfi, et al.’s (2021) results suggest that when matching number words and Arabic numerals in languages with inversion, there is an additional procedural step involved. Our findings suggest that this additional step includes the suppression of the incorrect numerical associations to arrive at the correct match or non-match decision. Interestingly, Clayton et al. (2020) recently also found evidence of an inversion effect for -teen numbers in English-speaking children, further generalising the inversion effect to specific numbers within a generally non-inverted number system.

It is also possible that participants who are highly proficient in a more transparent L2 may have two language-specific verbal mental representations concurrently activated when seeing a two-digit Arabic symbol, e.g., the English and the Dutch number-name representations. Bahnmueller et al. (2018) recently provided direct evidence for the fact that the linguistic structure of number words influences adults’ symbolic number processing. In other words, verbal representations of number words are concurrently activated even in tasks which do not require them. But so far this has only been examined within the context of a single language or with samples where participants’ multilingual profiles were not considered. In children, the inversion property can overload children’s working memory (Xenidou-Dervou et al., 2015). Similarly, Bahnmueller et al. (2018) demonstrated that linguistic influences in number-naming and cognitive control processes interact also in adulthood. Perhaps the concurrent activation of multiple verbal representations (one morpho-syntactically congruent and one incongruent with the structure of a two-digit Arabic symbol) may impose additional load on bilinguals’ cognitive abilities, especially for those who have formed a stronger association between a given two-digit number and its morpho-syntactic structure from another more transparent language besides the individuals’ L1.

To summarise, our study highlights that the way numbers are named matters in adulthood and that number word non-transparency affects adults’ numerical cognition. In particular, we found that the inversion property can thwart one’s decision on whether a two-digit Arabic symbol and a number word represent the same quantity. Our findings are also relevant to understanding the linguistic influences on number processing whether two-digit numbers are processed holistically or, more likely, in a hybrid fashion (Nuerk & Willmes, 2005). Our results suggest that non-transparency due to the inversion property is one factor that could influence the extent of holistic processing. Furthermore, our study accentuates the importance of future research in this domain to account for participants’ multilingual profiles. In general, bilingualism is an advantage (Antoniou, 2019), but, as evidenced in our study, bilingual proficiency can sometimes also complicate things when there is a conflict between number word morpho-syntactic structures across two languages (inversion vs no inversion). Given our diverse, multicultural societies, it is important to further understand how multilingualism dynamically interacts with linguistic influences in mathematical cognition.

## Supplemental Material

sj-docx-1-qjp-10.1177_17470218231181367 – Supplemental material for Multiple number-naming associations: How the inversion property affects adults’ two-digit number processing Supplemental material, sj-docx-1-qjp-10.1177_17470218231181367 for Multiple number-naming associations: How the inversion property affects adults’ two-digit number processing by Iro Xenidou-Dervou, Nienke van Atteveldt, Irina M Surducan, Bert Reynvoet, Serena Rossi and Camilla Gilmore in Quarterly Journal of Experimental Psychology
