# Supplementary material for: Multiple number-naming associations: How the inversion property affects adults’ two-digit number processing
Source: Q J Exp Psychol (Hove). 2023 Jul 7;77(4):856–72. doi: 10.1177/17470218231181367 (PMC10960323; doi:10.1177/17470218231181367)
Supplement: sj-docx-1-qjp-10.1177_17470218231181367 – Supplemental material for Multiple number-naming associations: How the inversion property affects adults’ two-digit number processing [file sj-docx-1-qjp-10.1177_17470218231181367.docx]

Supplementary Material for:

Multiple number-naming associations:

How the inversion property affects adults’ two-digit number processing.

Xenidou-Dervou Iro^1^, van Atteveldt Nienke^2^, Surducan Irina M^1^, Reynvoet Bert^3^, Rossi Serena^1^, Gilmore Camilla^1^

^1^Loughborough University, UK

^2^Vrije Universiteit Amsterdam, Netherlands

^3^KU Leuven, Belgium

**Corresponding Author:**

Xenidou-Dervou Iro

https://orcid.org/0000-0002-1511-6191

Loughborough University

Loughborough LE11 3TU

UK

I.Xenidou-Dervou@lboro.ac.uk

| **Supplementary Material Table A1.** Self-reported language history and proficiency (LEAP-Q questionnaire) | | | | | | | |
| --- | --- | --- | --- | --- | --- | --- | --- |
|  | **L1 history** | | | **L2 history** | | | |
| **Language history measures** | ***M*** | ***SD*** | **Range** | ***M*** | ***SD*** | **Range** | |
| Exposure to language (%) | 81.03 | 14.25 | 40-100 | 15.98 | 11.84 | 0-60 | |
| Chosen language to read (%) | 87.22 | 22.78 | 0-100 | 11.22 | 21.34 | 0-100 | |
| Chosen language to speak (%) | 90.13 | 18.64 | 20-100 | 7.36 | 13.91 | 0-70 | |
|  |  |  |  |  |  |  | |
| Age milestones (years) |  |  |  |  |  |  | |
| Began acquiring | 0.43 | 0.81 | 0-4 | 10.63 | 2.26 | 0-14 | |
| Became fluent | 3.98 | 1.29 | 1-8 | 13.60 | 2.59 | 0-18 | |
| Began reading | 5.17 | 1.09 | 3-8 | 12.50 | 1.82 | 8-20 | |
| Became fluent reading | 7.37 | 1.49 | 5-13 | 14.60 | 2.70 | 0-20 | |
|  |  |  |  |  |  |  | |
| Immersion duration (years) |  |  |  |  |  |  | |
| In a country | 30.83 | 14.90 | 19.33-65.33 | 0.11 | 0.24 | 0-1.16 | |
| In a family | 31.01 | 14.85 | 19.41-65.33 | 2.29 | 7.44 | 0-35 | |
| In a workplace/school | 29.75 | 15.29 | 16-65.33 | 1.46 | 2.86 | 0-12.5 | |
|  |  |  |  |  |  |  | |
| Proficiency speaking (0 - 10 scale) |  |  |  |  |  |  | |
| Speaking | 8.56 | 1.01 | 6-10 | 6.18 | 2.03 | 1-9 | |
| Understanding | 8.72 | 1.00 | 6-10 | 6.89 | 1.95 | 2-10 | |
| Reading | 8.51 | 1.15 | 6-10 | 6.43 | 2.09 | 1-10 | |
|  |  |  |  |  |  |  | |
| Contribution to language learning (0 - 10 scale) |  |  |  |  |  |  | |
| From family | 8.70 | 1.54 | 1-10 | 1.81 | 2.59 | 0-10 | |
| From friends | 7.36 | 1.61 | 1-10 | 2.94 | 2.95 | 0-10 | |
| From reading | 7.67 | 1.73 | 1-10 | 6.75 | 2.24 | 1-10 | |
| From TV | 6.31 | 1.84 | 1-10 | 6.65 | 2.16 | 0-10 | |
| From radio | 5.53 | 2.48 | 0-10 | 6.20 | 2.78 | 0-10 | |
| From classroom/self-instruction | 7.68 | 1.36 | 3-10 | 7.79 | 2.10 | 0-10 | |
|  |  |  |  |  |  |  | |
| Extent of language exposure (0 - 10 scale) |  |  |  |  |  |  | |
| Family | 8.53 | 1.54 | 3-10 | 1.41 | 2.64 | 0-10 | |
| Friends | 8.72 | 1.10 | 6-10 | 1.98 | 3.06 | 0-10 | |
| Reading | 7.44 | 2.09 | 2-10 | 6.12 | 2.81 | 0-10 | |
| TV | 6.67 | 2.29 | 0-10 | 6.62 | 2.23 | 0-10 | |
| Radio | 6.32 | 2.20 | 1-10 | 6.15 | 2.60 | 0-10 | |
| Classroom/self-instruction | 5.81 | 3.68 | 0-10 | 4.58 | 3.63 | 0-10 | |
|  |  |  |  |  |  |  | |
| Self-report of foreign accent (0 - 10 scale) |  |  |  |  |  |  | |
| Perceived by self | 0.31 | 0.94 | 0-5 | 4.34 | 2.92 | 0-10 | |
| Identified by others | 0.29 | 1.38 | 0-10 | 5.06 | 3.64 | 0-10 | |

**Supplementary Material Table A2.** Testing trials in the audiovisual matching task

|  |  | **Morpho-syntactic Congruency - Experimental Conditions** | | | | |  |
| --- | --- | --- | --- | --- | --- | --- | --- |
|  |  | Artificial | Traditional | Traditional | Traditional | Artificial | Traditional |
|  | **Arabic Number** | **Congruent Match** | **Congruent Match** | **Incongruent Match** | **Congruent Non-Match** | **Incongruent Non-Match** | **Incongruent Non-Match** |
| 1 | 49 | veertig en negen | forty-nine | negen en veertig | vier en negentig | negentig en vier | acht en twintig |
| 2 | 98 | negentig en acht | ninety-eight | acht en negetig | negen en tachtig | tachtig en negen | zes en vijftig |
| 3 | 56 | vijftig en zes | fifty-six | zes en vijftig | vijf en zestig | zestig en vijf | twee en dertig |
| 4 | 42 | veertig en twee | forty-two | twee en veertig | vier en twintig | twintig en vier | vier en twintig |
| 5 | 53 | vijftig en drie | fifty-three | drie en vijftig | vijf en dertig | dertig en vijf | drie en negentig |
| 6 | 48 | veertig en acht | forty-eight | acht en veertig | vier en tachtig | tachtig en vier | vier en tachtig |
| 7 | 29 | twintig en negen | twenty-nine | negen en twintig | twee en negentig | negentig en twee | een en vijftig |
| 8 | 28 | twintig en acht | twenty-eight | acht en twintig | twee en tachtig | tachtig en twee | negen en veertig |
| 9 | 84 | tachtig en vier | eighty-four | vier en tachtig | acht en veertig | veertig en acht | zes en vijftig |
| 10 | 39 | dertig en negen | thirty-nine | negen en dertig | drie en negetig | negentig en drie | zes en twintig |
| 11 | 63 | zestig en drie | sixty-three | drie en zestig | zes en dertig | dertig en zes | twee en veertig |
| 12 | 78 | zeventig en acht | seventy-eight | acht en zeventig | zeven en tachtig | tachtig en zeven | twee en vijftig |
| 13 | 24 | twintig en vier | twenty-four | vier en twintig | twee en veertig | veertig en twee | zes en dertig |
| 14 | 32 | dertig en twee | thirty-two | twee en dertig | drie en twintig | twintig en drie | acht en veertig |
| 15 | 54 | vijftig en vier | fifty-four | vier en vijftig | vijf en veertig | veertig en vijf | een en tachtig |
| 16 | 38 | dertig en acht | thirty-eight | acht en dertig | drie en tachtig | tachtig en drie | zeven en vijftig |
| 17 | 95 | negentig en vijf | ninety-five | vijf en negentig | negen en vijftig | vijftig en negen | zes en zeventig |
| 18 | 65 | zestig en vijf | sixty-five | vijf en zestig | zes en vijftig | vijftig en zes | twee en vijftig |
| 19 | 45 | veertig en vijf | forty-five | vijf en veertig | veer en vijftig | vijftig en veer | zes en dertig |
| 20 | 35 | dertig en vijf | thirty-five | vijf en dertig | drie en vijftig | vijftig en drie | acht en twintig |
| 21 | 76 | zeventig en zes | seventy-six | zes en zeventig | zeven en zestig | zestig en zeven | vijf en negentig |
| 22 | 68 | zestig en acht | sixty-eight | acht en zestig | zes en tachtig | tachtig en zes | vijf en tachtig |
| 23 | 52 | vijftig en twee | fifty-two | twee en vijftig | vijf en twintig | twintig en vijf | vijf en zestig |
| 24 | 36 | dertig en zes | thirty-six | zes en dertig | drie en zestig | zestig en drie | vijf en veertig |
